# *APLP2* gene polymorphisms are associated with high TC and LDL-C levels in Chinese population in Xinjiang, China

**DOI:** 10.1042/BSR20200357

**Published:** 2020-08-03

**Authors:** Jialin Abuzhalihan, Dilare Adi, Yong-Tao Wang, Yang Li, Yi-Ning Yang, Xiang Ma, Xiao-Mei Li, Xiang Xie, Fen Liu, Bang-Dang Chen, Min-Tao Gai, Zhen-Yan Fu, Yi-Tong Ma

**Affiliations:** 1 Department of Cardiology, First Affiliated Hospital of Xinjiang Medical University, Urumqi 830054, P.R. China; 2 Xinjiang Key Laboratory of Cardiovascular Disease Research, Urumqi 830054, P.R. China

**Keywords:** APLP2, lipid levels, polymorphism

## Abstract

Hyperlipidemia is one of the main risk factors for coronary artery disease (CAD). In the present study, we aimed to explore whether the single-nucleotide polymorphisms (SNPs) in amyloid precursor-like protein (APLP) 2 (*APLP2*) gene were associated with high lipid levels in Chinese population in Xinjiang, China. We recruited 1738 subjects (1187 men, 551 women) from the First Affiliated Hospital of Xinjiang Medical University, and genotyped three SNPs (rs2054247, rs3740881 and rs747180) of *APLP2* gene in all subjects by using the improved multiplex ligation detection reaction (iMLDR) method. Our study revealed that the rs2054247 SNP was associated with serum total cholesterol (TC), low-density lipoprotein cholesterol (LDL-C) levels, and high-density lipoprotein cholesterol (HDL-C) in additive model (all *P*<0.05). The rs747180 SNP was associated with serum TC and LDL-C levels in additive model (all *P*<0.05). Our study revealed that both rs2054247 and rs747180 SNPs of the *APLP2* gene were associated with high TC and LDL-C levels in Chinese subjects in Xinjiang.

## Introduction

Coronary artery disease (CAD), the leading cause of death, imposes immense health and economic burdens worldwide [1–3]. CAD is a complex disease with the involvement of multiple mechanisms, cell types, and is influenced by multiple risk factors such as diabetes, hyperlipidemia, smoking, chronic inflammation, immune diseases, hypertension, physical inactivity, and genetics [4–6]. Among these, hyperlipidemia plays a vital role in CAD development [7].

Dyslipidemia is a family of lipoprotein metabolism disorders manifested by elevated total cholesterol (TC), low-density lipoprotein cholesterol (LDL-C), triglycerides (TGs), and reduced high-density lipoprotein cholesterol (HDL-C) concentrations in the blood [8]. Studies showed that increased lipid levels result in vessel wall reactions, including endothelial dysfunction, smooth muscle cells proliferation, lipid accumulation, foam cell formation, and, finally, necrosis and plaque development, which were considered to increase the risk of CAD [9,10]. Genetic factors including single-nucleotide polymorphisms (SNPs) are the main risks that contribute to dyslipidemia.

Amyloid precursor-like protein (APLP) 2 (APLP2) belongs to the protein family that includes amyloid precursor protein (APP) and APLP1 in mammals, which has been associated with Alzheimer’s disease pathogenesis [11]. Consistent with this notion, APP and APLP2 are widely expressed in many tissues, whereas APLP1 is predominantly restricted to the neural cells [12–14]. APLP2 has been shown to regulate multiple cellular functions such as neurite outgrowth, axogenesis, corneal epithelial wound healing, cell adhesion, migration, and cholesterol homeostasis [15–17].

Previous studies have revealed that deletion of APLP2 may increase the development of atherosclerosis in animal models [18]. Researchers found that lack of *APLP2* gene led to increased catabolism of apoE/lipoprotein, and resulted in increased intracellular cholesterol levels [19]. However, little is known about *APLP2* gene in human population regarding plasma cholesterol levels, as well as the SNPs. Hence, we aimed to investigate the potential relationship between polymorphisms of human *APLP2* gene and serum cholesterol levels.

## Methods

### Ethical approval of the study protocol

The present study was approved by the Ethics Committee of the First Affiliated Hospital of Xinjiang Medical University, and was performed in accordance with the principles of the Declaration of Helsinki. Written informed consent was obtained from each participant, including explicit permission for the DNA analyses and the collection of relevant clinical data.

### Subjects

We randomly recruited 1826 subjects (1247 men, 579 women) from the First Affiliated Hospital of Xinjiang Medical University between August 2010 and October 2016. The present study included 1738 subjects (1187 men, 551 women) who had complete data on APLP2 genotype, and whole subjects are the residents in Xinjiang province. Exclusion criteria included those suffering from impaired malignancy, connective tissue disease, concomitant valvar heart disease, renal function, valvular disease or chronic inflammatory disease, pancreatic disease, fatty liver, cirrhosis, hepatitis. Moreover, subjects should also be free from thyroid disease, or any history of taking lipid-lowering drugs. Hypertension was defined as a systolic blood pressure ≥ 140 mmHg and/or a diastolic blood pressure ≥ 90 mmHg at least on two distinct occasions [20]. Diabetes mellitus was defined as two fasting plasma glucose (FPG) level ≥ 7.0 mmol/l [21]. The following information was collected: age, gender, hypertension, diabetes, TC, TG, HDL-C, and LDL-C. According to The 2016 Chinese Guidelines for the Management of Dyslipidemia in Adults [22]. high TC was defined as TC ≥ 6.22 mmol/l, high LDL-C was defined as LDL-C ≥ 4.14 mmol/l, low HDL-C was defined as HDL-C ≤ 1.04 mmol/l, and high TG was defined as TG ≥ 2.26 mmol/l. Blood samples were obtained from an antecubital vein into vacutainer tubes containing EDTA in the morning after an overnight fasting period. All the collected samples were transported on dry ice at prearranged intervals to Xinjiang Coronary Artery Disease VIP laboratory. The concentration of serum TC, TG, LDL-C, HDL-C, and FPG were measured by the Clinical Laboratory Department of the First Affiliated Hospital of Xinjiang Medical University with the biochemical analyzer (Dimension AR/AVL Clinical Chemistry System, Newark, NJ, U.S.A.) [23,24].

### Genotyping

Using Haploview 4.2 software and International HapMap Project website phase I & II database (http://www.hapmap.org), we obtained three tag SNPs of APLP2: SNP1 (rs2054247), SNP2 (rs3740881) and SNP3 (rs747180) by using minor allele frequency (MAF) ≥ 0.05 and linkage disequilibrium patterns with *r^2^* ≥ 0.8 as the cutoff. Blood samples were obtained from an antecubital vein into vacutainer tubes containing EDTA in the morning after an overnight fasting period. DNA was extracted from the peripheral blood leukocytes using a whole blood genome extraction kit (Beijing Biotech Corporation, Beijing, China). The SNP genotyping was performed using an improved multiplex ligation detection reaction (iMLDR) technique (Genesky Biotechnologies Inc., Shanghai, China). Genotyping was performed in a blinded fashion without knowledge of the participants’ clinical data, and a total of 10% of the genotyped samples were duplicated to monitor genotyping quality.

### Statistical analysis

The statistical analysis was performed using SPSS version 22.0 for Windows (SPSS Inc., Chicago, IL, U.S.A.). The Hardy–Weinberg equilibrium was assessed by Chi-square test. The continuous variables are shown as the means ± SD, and the differences between the male and the female subjects were assessed using an independent-sample *t* test. Differences in the categorical variables, such as the frequencies of smoking, drinking, hypertension, diabetes, and genotypes were analyzed using the chi-square test. Finally, after adjusting confounding variables, general linear model analysis was undertaken to test the association between APLP2 genotypes and lipid profile. In addition, a two-tailed *P*-value less than 0.05 was considered to be statistically significant.

## Results

### Clinical characteristics of subjects

The baseline characteristics of 1738 subjects (1187 men, 551 women) are shown in Table 1. The average age of the included subjects was 57.23 ± 10.96 years, The FPG levels and the prevalence of hypertension and diabetes were similar between males and females (all *P>*0.05). In females, plasma concentration of TC, LDL-C, and HDL-C is higher than males (all *P*<0.001). Further, in males, the TG levels and the prevalence of smoking and drinking were higher than females (all *P*<0.001).

**Table 1:** Clinical and metabolic characteristics of subjects

Risk factors	Total	Male	Female	*P*
Age (years)	57.23 ± 10.96	55.67 ± 11.05	60.58 ± 9.67	*<0.001*
Smoking, *n* (%)	634 (36.5%)	617 (52.0%)	17 (3.0%)	*<0.001*
Drinking, *n* (%)	402 (23.1%)	396 (33.4%)	6 (1.1%)	*<0.001*
Hypertension, *n* (%)	340 (19.6%)	225 (19.0%)	115 (20.9%)	0.363
Diabetes, *n* (%)	120 (6.9%)	88 (7.4%)	32 (5.8%)	0.263
TG, mean (SD)	1.86 ± 1.18	1.93 ± 1.28	1.69 ± 0.93	*<0.001*
TC, mean (SD)	3.98 ± 0.83	3.90 ± 0.83	4.16 ± 0.81	*<0.001*
HDL-C, mean (SD)	1.02 ± 0.31	0.96 ± 0.29	1.13 ± 0.33	*<0.001*
LDL-C, mean (SD)	2.47 ± 0.67	2.43 ± 0.68	2.56 ± 0.66	*<0.001*
FPG, mean (SD)	5.98 ± 2.47	6.00 ± 2.52	5.95 ± 2.36	0.723

Statistically significant values are in italics.

Data are presented as number of patients (%) or mean standard ± deviation.

### Distributions of genotype and allele in subjects

The distribution of genotypes and alleles for the three SNPs (rs2054247, rs3740881, and rs747180) of the *APLP2* gene is shown in Table 2. The genotype distributions of these SNPs met the Hardy–Weinberg equilibrium balance (all *P>*0.05). Our results showed that differences in SNP rs2054247 and SNP rs3740881 between males and females were not significant (all *P*>0.05; genotypes and additive model). However, our study revealed that the difference in SNP rs747180 distribution of genotype and additive model was significant between males and females for (*P*=0.013, *P*=0.012, respectively).

**Table 2:** Distribution of SNPs of *APLP2* gene in subjects

Genotype	Model		Total (*n*, %)	Male (*n*, %)	Female (*n*, %)	*P*
rs2054247	Genotypes	0	1203 (69.2)	832 (70.1)	371 (67.3)	0.341
		1	484 (27.8)	324 (27.3)	160 (29.0)	
		2	51 (2.9)	31 (2.6)	20 (3.6)	
	Additive model	GG+AA	1254 (72.2)	863 (72.7)	391 (71.0)	0.455
		GA	484 (27.8)	324 (27.3)	160 (29.0)	
rs3740881	Genotypes	0	1599 (92.0)	1095 (92.2)	504 (91.5)	0.501
		1	137 (7.9)	90 (7.6)	47 (8.5)	
		2	2 (0.1)	2 (0.2)	0 (0)	
	Additive model	GG+AA	1601 (92.1)	1097 (92.4)	504 (91.5)	0.504
		GA	137 (7.9)	90 (7.6)	47 (8.5)	
rs747180	Genotypes	2	1032 (59.4)	733 (61.8)	299 (54.3)	*0.013*
		1	586 (33.7)	377 (31.8)	209 (37.9)	
		0	120 (6.9)	77 (6.5)	43 (7.8)	
	Additive model	AA+GG	1152 (66.3)	810 (68.2)	342 (62.1)	*0.012*
		GA	586 (33.7)	377 (31.8)	209 (37.9)	

Statistically significant values are in italics.

GG:0, GA:1, AA:2.

### APLP2 genotypes and lipid levels

Our study revealed that the rs2054247 SNP was significantly related to serum TC and LDL-C levels in additive model before (*P*=0.008, *P*<0.001) and after multifactor adjustment of ethnicity, gender, age, smoking, drinking, hypertension, and diabetes (*P*=0.007, *P*<0.001; Table 3). In addition, the rs2054247 SNP was significantly correlated with serum HDL-C levels in additive model before and after multifactor adjustment (*P*<0.001, *P*=0.007; Table 3).

**Table 3:** Associations between rs2054247 and lipid parameters

	Homozygous for wild allele (G)	Heterozygous	Homozygous for rare allele (A)	*P*	Model 1		Model 2	
					β	*P* (Add)	β	*P* (Add)
TC (mmol/l)	3.93 ± 0.84	4.07 ± 0.78	4.33 ± 0.93	*<0.001*	0.063	*0.008*	0.063	*0.007*
LDL-C (mmol/l)	2.43 ± 0.68	2.56 ± 0.63	2.67 ± 0.69	*<0.001*	0.084	*<0.001*	0.085	*<0.001*
HDL-C (mmol/l)	1.00 ± 0.30	1.06 ± 0.34	1.03 ± 0.38	*0.004*	0.091	*<0.001*	0.062	*0.007*
TG (mmol/l)	1.87 ± 1.21	1.83 ± 1.13	1.80 ± 1.01	1	−0.015	0.519	−0.006	0.809

Statistically significant values are in italics.

Abbreviation: Add, additive model.

Model 1, Unadjusted model; Model 2, Analysis of covariance adjusted for ethnicity, gender, age, smoking, drinking, hypertension, and diabetes.

When analyzing three genotypes and additive model, we observed that the rs3740881 SNP was not associated with serum lipid levels before and after adjusting multifactor (all *P>*0.05; Table 4).

**Table 4:** Associations between rs3740881 and lipid parameters

	Homozygous for wild allele (G)	Heterozygous	Homozygous for rare allele (A)	*P*	Model 1		Model 2	
					β	*P* (Add)	Β	*P* (Add)
TC (mmol/l)	3.97 ± 0.83	4.11 ± 0.89	4.57 ± 1.40	0.488	0.043	0.075	0.038	0.109
LDL-C (mmol/l)	2.46 ± 0.67	2.57 ± 0.73	2.57 ± 0.73	0.764	0.043	0.071	0.041	0.085
HDL-C (mmol/l)	1.01 ± 0.31	1.05 ± 0.33	0.87 ± 0.13	1	0.035	0.139	0.014	0.536
TG (mmol/l)	1.86 ± 1.18	1.84 ± 1.26	1.11 ± 0.26	1	-0.005	0.843	0.006	0.814

Statistically significant values are in italics.

Abbreviation: Add, additive model.

Model 1, Unadjusted model; Model 2, Analysis of covariance adjusted for ethnicity, gender, age, smoking, drinking, hypertension, and diabetes.

Our study showed that the rs747180 SNP was associated with serum TC and LDL-C levels in additive model before (*P*=0.001, *P*<0.001) and after multifactor adjustment (*P*=0.001, *P*<0.001; Table 5). Furthermore, the rs747180 SNP was correlated with serum HDL-C levels in additive model (*P*=0.001). Nevertheless, after adjusting multifactor, there were not statistically significant differences.

**Table 5:** Associations between rs747180 and lipid parameters

	Homozygous for wild allele (A)	Heterozygous	Homozygous for rare allele (G)	*P*	Model 1		Model 2	
					β	*P* (Add)	β	*P* (Add)
TC (mmol/l)	3.90 ± 0.86	4.08 ± 0.78	4.24 ± 0.71	*<0.001*	0.079	*0.001*	0.079	*0.001*
LDL-C (mmol/l)	2.41 ± 0.70	2.56 ± 0.63	2.65 ± 0.55	*<0.001*	0.089	*<0.001*	0.087	*<0.001*
HDL-C (mmol/l)	0.99 ± 0.29	1.05 ± 0.34	1.05 ± 0.29	*0.004*	0.079	*0.001*	0.039	0.091
TG (mmol/l)	1.84 ± 1.21	1.89 ± 1.12	1.86 ± 1.28	1	0.022	0.355	0.035	0.148

Statistically significant values are in italics.

Abbreviation: Add, additive model.

Model 1: Unadjusted model; Model 2: Analysis of covariance adjusted for ethnicity, gender, age, smoking, drinking, hypertension and diabetes.

Finally, our study revealed that there was no significant association among the three SNPs and TG levels in participants (all *P>*0.05; Tables 3–5).

## Discussion

In the present study, we investigated the associations between three SNPs in the human *APLP2* gene and cholesterol levels among Chinese subjects in Xinjiang. This is the first attempt to study the common polymorphisms in *APLP2* gene and its association with four types of lipid parameters.

APP is a member of a family of homologous APLPs, including APLP1 and APLP2 [25]. The three proteins are highly homologous, sharing 38–51% amino acid sequence identity. APLP1 and APLP2 both undergo proteolytic processing similar to APP. APLP2, similar to APP, is ubiquitous and is expressed in many tissues, whereas APLP1 is found primarily in cells of the nervous system [26–28]. Studies in APP and APLP1/2 knockout (KO) mice suggest functional differences among the three family members and, significantly, a particular importance for APLP2 in the physiology of the cholesterol metabolism and coronary artery atherosclerosis [29]. However, the association between *APLP2* gene polymorphisms and serum lipid levels was poor.

The relationship between cholesterol levels and APLP2 was first studied by Liu et al. [19]. They investigated apoE and cholesterol levels in mouse embryonic fibroblasts (MEFs) of wildtype (WT), APP-KO, or APP and APLP2 double-KOs (APP/APLP2-DKO). The APP-KO and APP/APLP2-DKO cells displayed a significant decrease in apoE levels and a concomitant increase in cholesterol levels compared with WT controls. Additionally, they measured these changes *in vivo*, brain apoE, and cholesterol levels in WT, APP-KO, and APP/APLP2-DKO mouse brain. ApoE levels were decreased by 50% in APP-KO and further decreased in APP/APLP2-DKO when compared with WT littermate controls. A corresponding increase in cholesterol levels was also observed in APP-KO and APP/APLP2-DKO mouse brain when compared with WT controls.

Our study showed that rs2054247 was significantly associated with plasma TC and LDL-C levels in additive model. Such association remained significant after multivariate adjustment. As is known to all, hyperlipidemia is the key factor that contributes to the development of atherosclerotic cardiovascular disease (CVD), such as CAD. In present study, we have found that rs2054247 of *APLP2* gene was associated with hyperlipidemia among Chinese subjects, and thus assumed AA genotype of rs2054247 might be a risk genetic marker for coronary heart disease (CHD). Together, these results might provide convincing evidence for assuming people who carry A allele may have higher probabilities of suffering from atherosclerotic CVD than people who carry G allele of rs2054247 of *APLP2* gene.

We further observed that rs747180 was also significantly associated with plasma TC and LDL-C levels in additive model before and after adjustment. Individuals with GG genotype of rs747180 had higher prevalence of plasma TC and LDL-C levels, indicating that G allele of rs747180 of APLP2 gene may be a risk genetic marker for CVD.

Finally, our study did not show a remarkable difference between TG levels and three kinds of SNPs of *APLP2* gene. This might indicate that *APLP2* gene does not have as strong correlation with TG concentration as it does with cholesterol concentration. Nevertheless, the relationship between TG concentration and *APLP2* gene needs further study.

There are several limitations in our study. First, our subjects were limited to the First Affiliated Hospital of Xinjiang Medical University, and we only drew conclusions based on the present observational association study. Second, the present study lacked functional validation. Additional studies need to be undertaken to clarify the underlying molecular mechanism that associate the *APLP2* gene polymorphisms with hyperlipidemia.

## Conclusions

In conclusion, our study revealed that rs2054247 and rs747180 of *APLP2* gene were associated with high TC and LDL-C levels in Chinese subjects in Xinjiang. Subjects with AA/GA genotype of rs2054247 may have higher risks from suffering hyperlipidemia. In the same way, subjects with GG/GA genotype of rs747180 were associated with an increased risk of hyperlipidemia.

## Data Availability

The data will not be shared, since part of the data is being reused by another study.

## Abbreviations

APLP: amyloid precursor-like protein
APP: amyloid precursor protein
CAD: coronary artery disease
CVD: cardiovascular disease
DKO: double-knockout
FPG: fasting plasma glucose
HDL-C: high-density lipoprotein cholesterol
KO: knockout
LDL-C: low-density lipoprotein cholesterol
SNP: single-nucleotide polymorphism
TC: total cholesterol
TG: triglyceride

## References

[1] Benjamin E.J., Muntner P., Alonso A. et al. (2019) Heart Disease and Stroke Statistics-2019 Update: a report from the American Heart Association. Circulation 139, e56–e528 30700139 10.1161/CIR.0000000000000659

[2] Miró Ò., Peacock F.W., McMurray J.J. et al. (2017) European Society of Cardiology-Acute Cardiovascular Care Association position paper on safe discharge of acute heart failure patients from the emergency department. Eur. Heart J. Acute Cardiovasc. Care 6, 311–320 26900163 10.1177/2048872616633853 PMC4992666

[3] Erbel R., Aboyabs V., Boilieu C. et al. (2014) 2014 ESC Guidelines on the diagnosis and treatment of aortic diseases: Document covering acute and chronic aortic diseases of the thoracic and abdominal aorta of the adult. The Task Force for the Diagnosis and Treatment of Aortic Diseases of the European Society of Cardiology (ESC). Eur. Heart J. 35, 2873–2926 25173340 10.1093/eurheartj/ehu281

[4] Piepoli M.F., Hoes A.W., Agewall S. et al. (2017) 2016 European guidelines on cardiovascular disease prevention in clinical practice. The Sixth Joint Task Force of the European Society of Cardiology and Other Societies on Cardiovascular Disease Prevention in Clinical Practice (constituted by representatives of 10 societies and by invited experts. Developed with the special contribution of the European Association for Cardiovascular Prevention & Rehabilitation. G. Ital. Cardiol. 18, 547–612 28714997

[5] Kwak B.R., Bäck M., Bochaton-Piallat M.-L. et al. (2014) Biomechanical factors in atherosclerosis: mechanisms and clinical implications. Eur. Heart J. 35, 3013–3020, 3020a–3020d 10.1093/eurheartj/ehu353 25230814 PMC4810806

[6] Zhao Y., Chen J., Freudenberg J.M. et al. (2016) Network-based identification and prioritization of key regulators of coronary artery disease loci. Arterioscler. Thromb. Vasc. Biol. 36, 928–941 26966275 10.1161/ATVBAHA.115.306725 PMC5576868

[7] Smith D.A. (2019) Review: In dyslipidemia or atherosclerotic CVD, alirocumab and evolocumab vs control each reduce MI and stroke. Ann. Intern. Med. 171, JC56 10.7326/ACPJ201911190-056 31739339

[8] Xiao J., Luo J., Ho A. et al. (2019) Cholesterol transport through the peroxisome-ER membrane contacts tethered by PI(4,5)P and extended synaptotagmins. Sci. China Life Sci. 62, 1117–1135 31144242 10.1007/s11427-019-9569-9

[9] Chu B.B., Liao Y.-C., Qi W. et al. (2015) Cholesterol transport through lysosome-peroxisome membrane contacts. Cell 161, 291–306 25860611 10.1016/j.cell.2015.02.019

[10] Park G.-M., Lee Y., Won K.-B. et al. (2019) High HDL-C levels reduce the risk of obstructive coronary artery disease in asymptomatic diabetics who achieved optimal glycemic control. Sci. Rep. 9, 15306 31654036 10.1038/s41598-019-51732-6 PMC6814721

[11] Heber S., Herms J., Gajic V. et al. (2000) Mice with combined gene knock-outs reveal essential and partially redundant functions of amyloid precursor protein family members. J. Neurosci. 20, 7951–7963 10.1523/JNEUROSCI.20-21-07951.2000 11050115 PMC6772747

[12] Sprecher C.A., Grant F.J., Grimm G. et al. (1993) Molecular cloning of the cDNA for a human amyloid precursor protein homolog: evidence for a multigene family. Biochemistry 32, 4481–4486 10.1021/bi00068a002 8485127

[13] Wasco W., Gurubhagavatula S., Paradis M.D. et al. (1993) Isolation and characterization of APLP2 encoding a homologue of the Alzheimer's associated amyloid beta protein precursor. Nat. Genet. 5, 95–100 10.1038/ng0993-95 8220435

[14] Walsh D.M., Minogue A.M., Sala Frigerio C. et al. (2007) The APP family of proteins: similarities and differences. Biochem. Soc. Trans. 35, 416–420 10.1042/BST0350416 17371289

[15] Cappai R., Mok S.S., Galatis D. et al. (1999) Recombinant human amyloid precursor-like protein 2 (APLP2) expressed in the yeast Pichia pastoris can stimulate neurite outgrowth. FEBS Lett. 442, 95–98 10.1016/S0014-5793(98)01635-4 9923612

[16] Guo J., Thinakaran G., Guo Y. et al. (1998) A role for amyloid precursor-like protein 2 in corneal epithelial wound healing. Invest. Ophthalmol. Vis. Sci. 39, 292–300 9477985

[17] Li X.F., Thinakaran G., Sisodia S.S. et al. (1999) Amyloid precursor-like protein 2 promotes cell migration toward fibronectin and collagen IV. J. Biol. Chem. 274, 27249–27256 10.1074/jbc.274.38.27249 10480944

[18] Grimm M.O.W., Grimm H.S., Pätzold A.J. et al. (2005) Regulation of cholesterol and sphingomyelin metabolism by amyloid-beta and presenilin. Nat. Cell Biol. 7, 1118–1123 10.1038/ncb1313 16227967

[19] Liu Q., Zerbinatti C.V., Zhang J. et al. (2007) Amyloid precursor protein regulates brain apolipoprotein E and cholesterol metabolism through lipoprotein receptor LRP1. Neuron 56, 66–78 17920016 10.1016/j.neuron.2007.08.008 PMC2045076

[20] Gidding S.S., Whelton P.K., Carey R.M. et al. (2019) Writing a trustworthy hypertension guideline. J. Am. Coll. Cardiol. 74, 2424–2427 10.1016/j.jacc.2019.09.008 31699283

[21] Shen J.I., Nicholas S.B., Williams S. et al. (2019) Evidence for and against ACC/AHA 2017 Guideline for target systolic blood pressure of < 130 mmHg in persons with Type 2 Diabetes. Curr. Cardiol. Rep. 21, 149 31760494 10.1007/s11886-019-1251-4 PMC6984818

[22] Pan S. et al. (2013) Appropriate body mass index and waist circumference cutoffs for categorization of overweight and central adiposity among Uighur adults in Xinjiang. PLoS ONE 8, e80185 10.1371/journal.pone.0080185 24244645 PMC3820640

[23] Ma J., Zhou Y. et al. (2019) Association between urinary polycyclic aromatic hydrocarbon metabolites and dyslipidemias in the Chinese general population: a cross-sectional study. Environ. Pollut. 245, 89–97 10.1016/j.envpol.2018.10.134 30415036

[24] Joint committee for guideline revision (2018) 2016 Chinese guidelines for the management of dyslipidemia in adults. J. Geriatr. Cardiol. 15, 1–29 29434622 10.11909/j.issn.1671-5411.2018.01.011 PMC5803534

[25] Herms J., Anliker B., Heber S. et al. (2004) Cortical dysplasia resembling human type 2 lissencephaly in mice lacking all three APP family members. EMBO J. 23, 4106–4015 15385965 10.1038/sj.emboj.7600390 PMC524337

[26] Thinakaran G., Kitt C.A., Roskams A.J. et al. (1995) Distribution of an APP homolog, APLP2, in the mouse olfactory system: a potential role for APLP2 in axogenesis. J. Neurosci. 15, 6314–6326 10.1523/JNEUROSCI.15-10-06314.1995 7472397 PMC6577992

[27] Tan J.Z.A. and Gleeson P.A. (2019) The trans-Golgi network is a major site for α-secretase processing of amyloid precursor protein in primary neurons. J. Biol. Chem. 294, 1618–1631 30545942 10.1074/jbc.RA118.005222 PMC6364769

[28] Tuli A., Sharma M., Capek H.L. et al. (2009) Mechanism for amyloid precursor-like protein 2 enhancement of major histocompatibility complex class I molecule degradation. J. Biol. Chem. 284, 34296–34307 19808674 10.1074/jbc.M109.039727 PMC2797198

[29] von Koch C.S., Zheng H., Chen H. et al. (1997) Generation of APLP2 KO mice and early postnatal lethality in APLP2/APP double KO mice. Neurobiol. Aging. 18, 661–669 9461064 10.1016/s0197-4580(97)00151-6

